# Localized Corrosion Occurrence in Low-Carbon Steel Pipe Caused by Microstructural Inhomogeneity

**DOI:** 10.3390/ma15051870

**Published:** 2022-03-02

**Authors:** Yun-Ho Lee, Geon-Il Kim, Kyung-Min Kim, Sang-Jin Ko, Woo-Cheol Kim, Jung-Gu Kim

**Affiliations:** 1 School of Advanced Materials Science and Engineering, Sungkyunkwan University (SKKU), Suwon 16419, Korea; yunho0228@naver.com (Y.-H.L.); geonil1996@naver.com (G.-I.K.); kkm2628@gmail.com (K.-M.K.); tkdwls1315@naver.com (S.-J.K.); 2 Technical Efficiency Research Team, Korea District Heating Corporation, 92 Gigok-ro, Yongin 06340, Korea; kwc7777@kdhc.co.kr

**Keywords:** failure analysis, low-carbon steel pipe, pitting corrosion, aluminum inclusions, pearlite inhomogeneity

## Abstract

In this study, the cause of failure of a low-carbon steel pipe meeting standard KS D 3562 (ASTM A135), in a district heating system was investigated. After 6 years of operation, the pipe failed prematurely due to pitting corrosion, which occurred both inside and outside of the pipe. Pitting corrosion occurred more prominently outside the pipe than inside, where water quality is controlled. The analysis indicated that the pipe failure occurred due to aluminum inclusions and the presence of a pearlite inhomogeneous phase fraction. Crevice corrosion occurred in the vicinity around the aluminum inclusions, causing localized corrosion. In the large pearlite fraction region, cementite in the pearlite acted as a cathode to promote dissolution of surrounding ferrite. Therefore, in the groundwater environment outside of the pipe, localized corrosion occurred due to crevice corrosion by aluminum inclusions, and localized corrosion was accelerated by the large fraction of pearlite around the aluminum inclusions, leading to pipe failure.

## 1. Introduction

District heating (DH) systems produce heat that is used to provide steam and hot water to the residents of large cities [1,2]. These heating systems provide higher thermal efficiency and lower heating costs than small private boiler units [3]. The steam and hot water that are transported in DH systems expose pipes to corrosion risks. Failure of system pipes due to corrosion reduces thermal efficiency and negatively impacts the system’s cost and reliability [1]. As such, failure analyses and pipe corrosion prevention are important for the maintenance of DH systems.

Low-carbon steel has been widely used as a pipe material in various industrial plants such as those that manage oil, gas, and water, as well as in DH systems [1,4,5,6,7,8]. Several studies have been conducted on the failure of low-carbon steel pipes. Kim et al. reported that crack propagation occurs due to stress concentrations and high hydrogen susceptibility in the weld zone (WZ) and heat-affected zone caused by poor welding, resulting in the failure of low-carbon steel pipe [1]. Lee et al. reported that the failure of low-carbon steel pipes are caused by stress corrosion cracking due to chloride presence and residual stress in the WZ [5]. Heyes et al. observed the fatigue cracking as a result of oxygen-induced pitting in the pipe [9]. Various other failure types and mechanisms can be found in available literatures [10].

In most studies, the failure of low-carbon steel pipe occurs in the form of stress corrosion cracking (SCC) or corrosion fatigue cracking (CFC) when stress is applied. However, the failure shown in this study was due to pitting corrosion. Low-carbon steel is not passivated as stainless steel is, and corrosion generally occurs uniformly over the pipe. Pitting corrosion is rarely observed on the low-carbon steel pipe [11]. Therefore, it is necessary to investigate the cause of pitting corrosion of low-carbon steel pipe to prevent specific corrosion and sudden leakage.

This study analyzes the failure of a low-carbon steel pipe due to corrosion, which has not been reported in the actual use of low-carbon steel pipe. Compliance with material specification was evaluated using inductively coupled plasma atomic emission spectroscopy (ICP-AES) component analysis. The cause of the failure was then analyzed through visual inspection, optical microscopy (OM), metallographic examination, scanning electron microscopy (SEM) with energy dispersive spectroscopy (EDS), electron probe microanalyzer (EPMA) and atomic force microscopy (AFM). Furthermore, the electrochemical properties of the failed low-carbon steel were evaluated using the potentiodynamic polarization and galvanostatic polarization tests.

## 2. Materials and Methods

### 2.1. Description of the Pipeline

**Figure 1:**
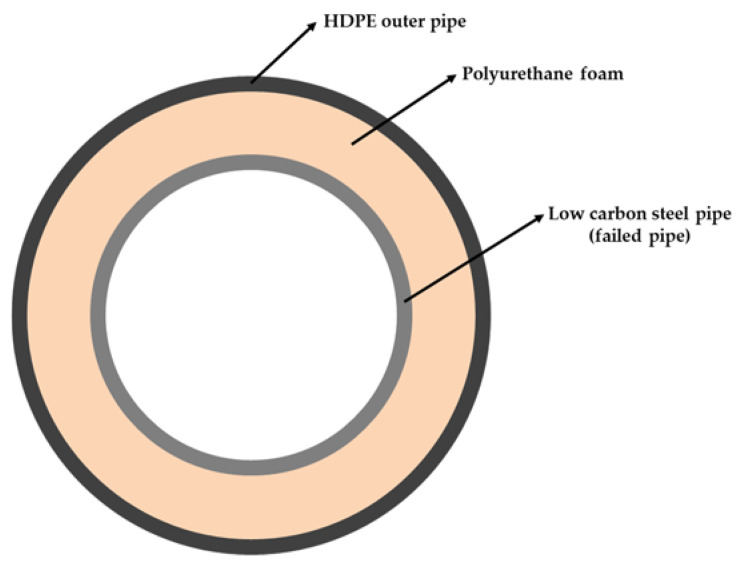
A schematic illustration of the heat transport pipe used in a DH system.

Before conducting the failure analysis of the low-carbon steel pipe, the specifications and environment were investigated. According to the user’s description, the failed low-carbon steel pipe transported hot water through underground pipeline in DH systems. The low-carbon steel pipe satisfied standard KS D 3562 (ASTM A135, Electric-Resistance-Welded Steel Pipe Grade A) [1,12,13,14]. The outer diameter of the pipe was 457.2 mm with a wall thickness of 6.4 mm. As shown in Figure 1, the buried pipe is surrounded by polyurethane foam as a heat insulator and a high-density polyethylene (HDPE) pipe as an outer casing. Internal corrosion can occur inside of the pipe caused by the transported water in the DH system. External corrosion can occur on the exterior surface of the pipe due to degradation of the HDPE pipe and subsequent penetration of groundwater [15,16]. Table 1 and Table 2 present the chemical composition of the DH water inside of the pipe and the synthetic groundwater outside of the pipe, respectively [15,17]. In DH systems, the design service life of a low-carbon steel pipe is 40 years; however, the pipe observed in this study failed after only 6 years of operation [1].

### 2.2. Metallurgical Analyses

In this study, it was investigated whether the material used for the pipe satisfies the KS D 3562 standard through chemical composition analysis. The shape of the failed pipe was visually investigated via OM. The metallographic examination was performed to confirm the microstructure uniformity of the pipe metal. To investigate the microstructure of the pipe metal, the specimen was polished using a 1-µm diamond suspension, and thereafter etched by a 2% Nital etching solution for 20 s [1,4]. The volume fractions of pearlite and ferrite phases in the microstructure according to the location of corrosion in the failed pipe were measured by OM using Image J software (version 1.8.0) [18]. For the OM image, the fraction of each phase was calculated by counting the number of pixels in ferrite and pearlite and dividing by the total number of pixels. For microanalysis of the microstructure, topography and surface potential were measured using AFM and kelvin probe force microscopy (KPFM), a mode of AFM. AFM measurements were performed using a commercial AFM system (NX10, Park Systems, Suwon, Korea). KPFM measurements were performed using a conductive Pt/Cr coated tip (Multi75E-G, BudgetSensors, Sofia, Bulgaria) in lift mode with a tip-to-sample distance of 20 nm, and an AC modulation voltage of 2V_rms_ at 17 kHz. Measurements were performed at 10 µm × 10 µm and 2 µm × 2 µm, respectively. The fracture properties were analyzed using SEM/EDS (SEM-7800F Prime, JEOL Ltd., Tokyo, Japan) and EPMA (JXA-8530F, JEOL Ltd., Tokyo, Japan). Prior to SEM/EDS and EPMA analyses, specimens were pickled, polished with 1000-grit size silicon carbide paper, and then rinsed with deionized water and cleaned with ethanol.

### 2.3. Electrochemical Tests

Potentiodynamic polarization and galvanostatic polarization tests were performed using a VSP 300 (Bio-Logic SAS, Seyssinet-Pariset, France). To conduct these electrochemical tests, a three-electrode system comprising low-carbon steel pipe specimen taken from the failed pipe as the working electrode (WE), two pure graphite rods as the counter electrodes (CE), and a saturated calomel electrode (SCE) with a Luggin capillary as the reference electrode (RE) was used. To confirm the cause of the pitting corrosion, specimens were prepared for the pitting corrosion part (specimen A) and the uniform corrosion part (specimen B) of the failed pipe. For electrochemical tests, all specimens were polished with a 1000-grit silicon carbide paper, rinsed with ethanol, and dried with nitrogen gas. The area of all specimens was controlled to a size of 1 cm^2^ using a sealant. A groundwater solution was used for the electrochemical tests (Table 2) because it was more corrosive to the pipe. The temperature was maintained at 60 °C to reflect the temperature of the distinct heating system [2,14,16]. Before conducting the electrochemical tests, the WE was immersed in the test solution for 6 h to obtain a stable open-circuit potential (OCP) as the corrosion potential (E_corr_). The potentiodynamic polarization tests were conducted using a potential sweep of 0.01 mV/s from −0.25 V vs. E_corr_ to 1.6 V vs. E_corr_. The galvanostatic polarization tests were performed at 5 mA/cm^2^ to accelerate corrosion. The total amount of coulombic charge was 143.86 mAh, which is equivalent to 6 months of uniform corrosion in specimen B. To evaluate the same amount of corrosion, the same coulombic charge was applied to both specimens.

## 3. Results and Discussion

### 3.1. Chemical Composition Analysis of the Low-Carbon Steel Pipe

Table 3 shows the chemical composition analysis of the low-carbon steel pipe material, as well as the KS D 3562 standard. It was confirmed that the composition of the failed pipe material complied with the KS D 3562 standard. A key finding of note was the detection of a small amount of Al component in the failed pipe, where KS D 3562 does not dictate any Al component.

### 3.2. Visual and Macroscopic Inspections

**Figure 2:**
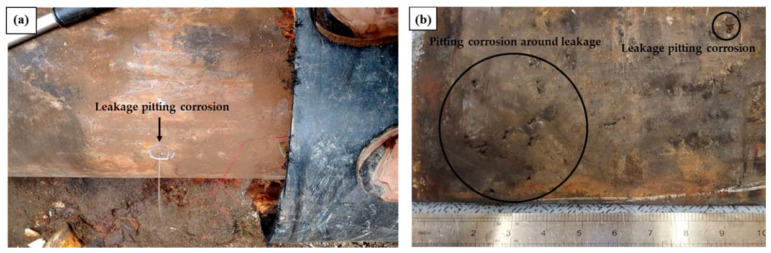
Photographs of the failed pipe: (**a**) water leakage resulting from pitting in the failed pipe, (**b**) external surface of the failed pipe around the leakage area.

**Figure 3:**
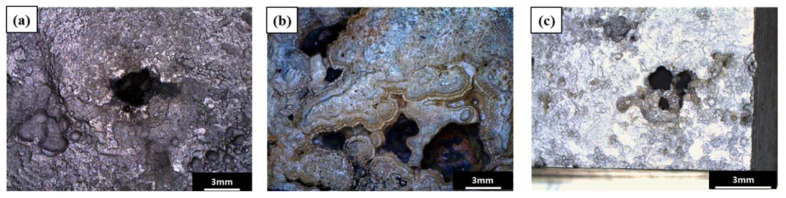
Surface image of the pitting part: (**a**) pitting corrosion area of the leakage (the outside of the pipe), (**b**) pitting corrosion area near the leakage (the outside of the pipe), and (**c**) pitting corrosion area near the leakage (the inside of the pipe).

**Figure 4:**
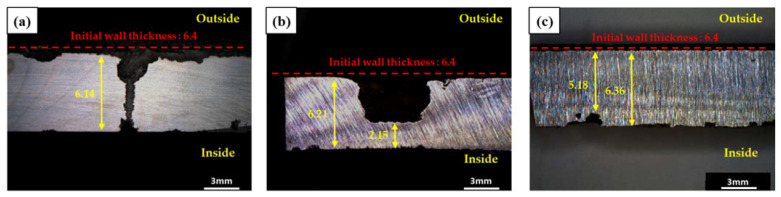
Cross-sectional images of the pitting part: (**a**) pitting corrosion where the leakage occurred, (**b**) pitting corrosion near the leakage, and (**c**) pitting corrosion near the leakage.

Based on visual inspection of the failed pipe, leakage occurred in the form of pitting, and the severe pitting corrosion occurred near the part of the pipe where the leaking occurred (Figure 2). Figure 3 shows surface images of the pitting corrosion part where leakage occurred, as well as pitting corrosion near the leakage. Figure 4 shows cross-sectional images of the pitting corrosion part where the leakage occurred and the pitting corrosion near the leakage. As stated previously, pitting corrosion occurred on both the exterior and interior of the failed pipe wall. However, since low-carbon steel is a material that does not have passivation, pitting corrosion cannot occur due to the failure of passivation. In low-carbon steel, localized corrosion can be caused by crevice corrosion by specific inclusions, galvanic corrosion between dissimilar metals, and under deposit corrosion (UDC) by solid particles (sand, debris, and iron oxides) [14,19]. In other words, it is necessary to investigate the above possibilities openly.

Moreover, the depth of the pitting corrosion on the exterior of the failed pipe was significantly deeper than the interior. It is considered to have been due to the difference between the environment of the inside and outside of the pipe (Table 1 and Table 2). In addition, to reduce the risk of corrosion, the DH water that flowed through the pipe was maintained at a dissolved oxygen level below 200 ppm through periodic water quality management [15]. In low-carbon steel, localized corrosion hardly occurs when the concentration of dissolved oxygen is low [20]. In addition, in the case of the exterior of the pipe, localized corrosion may be further accelerated due to the non-uniform distribution of groundwater penetrating into the heat insulator. Therefore, it is considered that the corrosion perforation of the pipeline is induced by the external corrosion.

### 3.3. Metallographic Examinations and Atomic Force Microscopy

**Figure 5:**
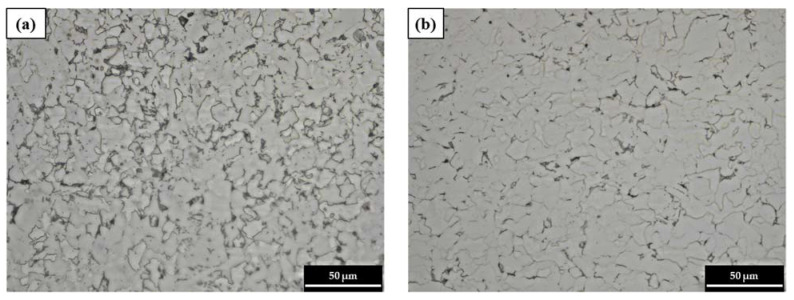
The optical microstructures of specimens: (**a**) specimen A (pitting corrosion region), and (**b**) specimen B (uniform corrosion region).

Figure 5 shows the microstructure of the pitting corrosion region (specimen A) and the uniform corrosion region (specimen B) of the failed pipe. The darker-colored section is pearlite, which is a layered structure composed of ferrite and cementite, and the brighter-colored section is ferrite [21]. Figure 5 and Table 4 show that there is a larger fraction of pearlite in specimen A than in specimen B. Pearlite is susceptible to corrosion as its constituent phases, ferrite and cementite, have dissimilar electrochemical potentials that cause microgalvanic corrosion when exposed to corrosive electrolytes [21,22,23].

**Figure 6:**
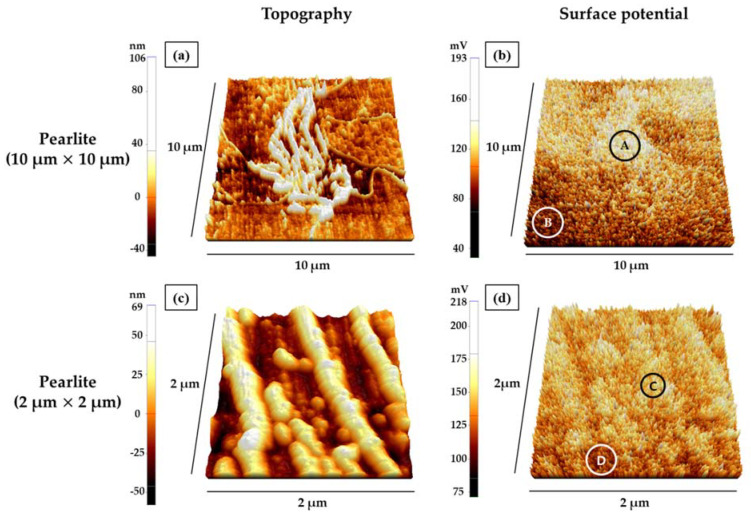
Topography and surface potential of pearlite (specimen A): (**a**) topography of pearlite, (**b**) surface potential of pearlite, (**c**) topography inside of pearlite, and (**d**) surface potential inside of pearlite.

Figure 6 shows the AFM and KPFM analyses of the pearlite section of the failed pipe. Figure 6a,b show the correlation between pearlite and the surrounding pro-eutectoid ferrite by measuring topology and surface potential within a size of 10 μm × 10 μm, respectively. In Figure 6a, pearlite has a lamella structure, and the surface potential of pearlite (A site) is approximately 44.4 mV higher than that of the surrounding pro-eutectoid ferrite (B site) in Figure 6b and Table 5. This indicates that the corrosion of pro-eutectoid ferrite is locally accelerated by microgalvanic corrosion between pearlite and the surrounding pro-eutectoid ferrite [19,24]. Figure 6c,d show the correlation between cementite and ferrite in the pearlite by measuring topology and surface potential within a size of 2 μm × 2 μm, respectively. Figure 6c shows the lamella structure of pearlite, indicating that the bright protrusions are cementite while the dark region is the ferrite [25]. This is because the corrosion of ferrite was more corroded than that of cementite during the etching process using 2% Nital etching solution. Figure 6d and Table 5 show that the surface potential of cementite in the pearlite (C site) is approximately 34.78 mV higher than that of ferrite in the pearlite (D site). Thus, the corrosion of ferrite is locally accelerated by microgalvanic corrosion between ferrite and cementite in the pearlite [19,24].

Therefore, the inhomogeneity of the microstructure forces a difference in corrosion rate. A pipe section exhibiting pitting corrosion with a lot of pearlite experiences severe corrosion, whereas a uniform corrosion section of the failed pipe with a low pearlite phase incurs only minor thickness reduction.

### 3.4. Microscopic Analyses

**Figure 7:**
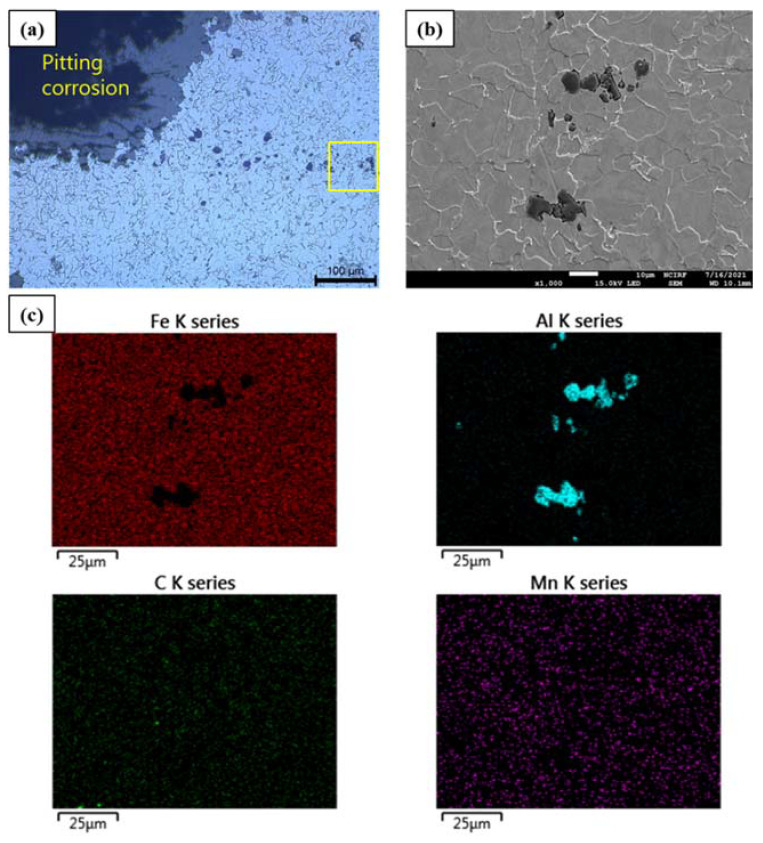
The OM, and SEM/EDS analyses of the pitting corrosion near the location of the leakage; (**a**) OM analysis, (**b**) SEM analysis (yellow box), and (**c**) EDS mapping analysis (yellow box).

To identify the cause of severe pitting corrosion, microscopic analyses were performed on the pitting corrosion near the leakage area using SEM/EDS and EPMA (Figure 7). Figure 7a,b showed several inclusions around the pitting corrosion site. The EDS elemental mapping of the inclusions in Figure 7c reveals that the particle is primarily composed of Al. The size range of these Al inclusions is 10–20 µm. In the chemical composition analysis, 0.04% Al was contained in the failed pipe, as shown in Table 3.

Al inclusions were considered to have been the result of Al use as a deoxidizer in the steelmaking process [26]. Al is known to have uniform distribution and fine particle size (less than 2 µm) upon proper heat treatment; accordingly, inclusions typically have little influence on corrosion behavior [27]. However, the failed pipe appeared to have larger-sized Al inclusions as well as an uneven distribution compared with the results in the literature [27,28]. This may have been the result of improper or incomplete homogenization during the steelmaking process. Furthermore, during the pipe manufacturing process, microcrevices formed at matrix–inclusion interfaces due to dissimilarities in the strain values and thermal expansion coefficients, which may have caused crevice corrosion, thereby accelerating localized corrosion [19,29].

**Figure 8:**
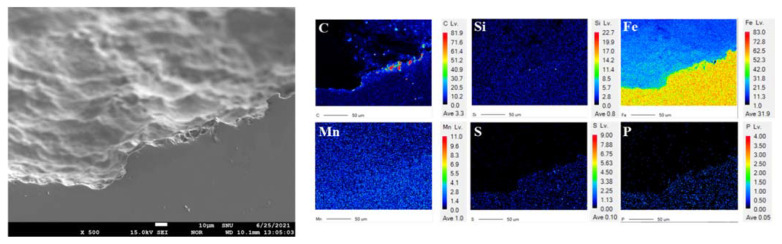
The EPMA analysis of a cross section where pitting corrosion occurred in the failed pipe.

Figure 8 shows the EPMA analysis of a cross section where pitting corrosion occurred in the failed pipe. Carbon agglomerations were partially observed in the pitting corrosion part. It appeared that the pitting corrosion region had a larger pearlite fraction than the uniform corrosion region in the failed pipe. Accordingly, the carbon agglomerations occurred due to the high fractional presence of cementite in the pearlite. As shown in the AFM and KPFM analyses results (Figure 6 and Table 5), when there are many pearlite phases, the material is vulnerable to corrosion due to microgalvanic corrosion between pearlite and surrounding pro-eutectoid ferrite, ferrite, and cementite [19,24,30]. The ferrite is corroded locally by microgalvanic corrosion around cementite.

### 3.5. Open-Circuit Potential Measurement

**Figure 9:**
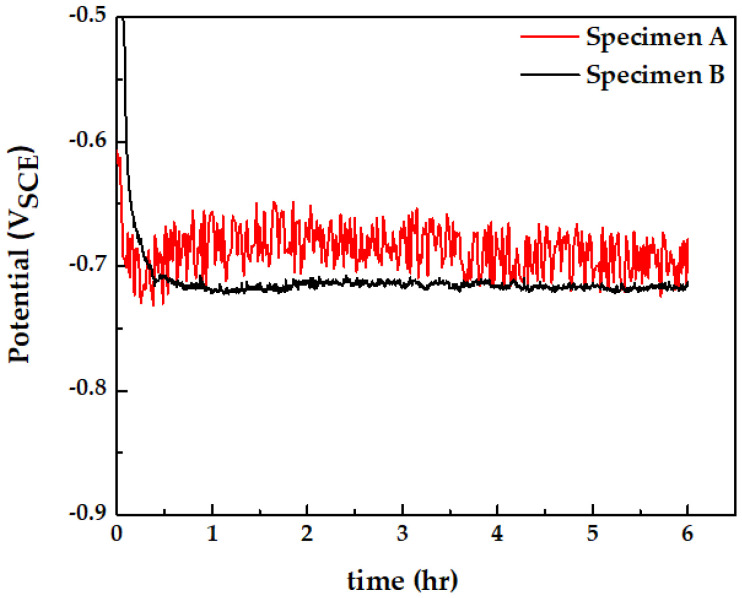
Open-circuit potential of specimen A (pitting corrosion region) and specimen B (uniform corrosion region) with immersion time in the groundwater at 60 °C.

Figure 9 shows the open-circuit potential (OCP) of specimen A and specimen B in the groundwater solution. Specimen A had a higher E_corr_ than specimen B, and had a relatively large potential fluctuation of approximately 50 mV. Specimen A has a higher E_corr_ due to a larger fraction of pearlite, which has a relatively noble potential compared to ferrite. Pearlite has a higher E_corr_ than ferrite due to an increase in cathodic sites that cause oxygen reduction (O_2_ + 2H_2_O + 4e = 4OH^−^) [31,32]. In addition, due to the higher corrosion activity caused by larger fraction of pearlite and presence of Al inclusions, OCP fluctuation is shown on specimen A [33,34].

### 3.6. Potentiodynamic Polarization Test

**Figure 10:**
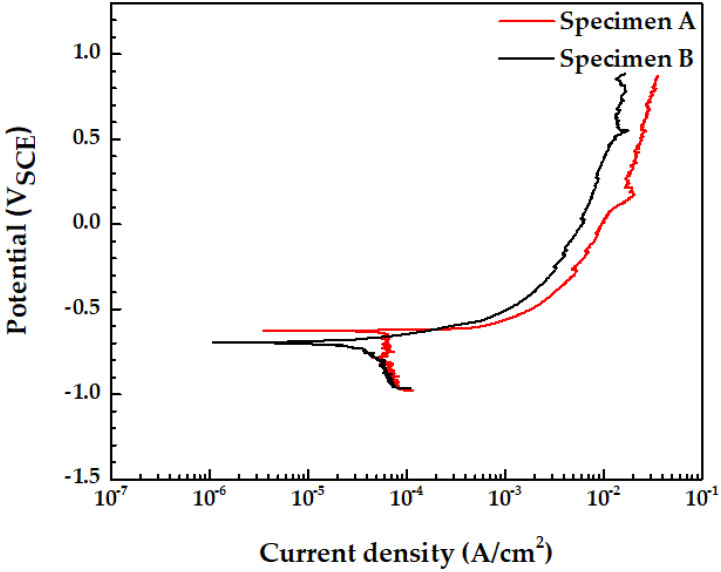
Potentiodynamic polarization curves of specimen A (pitting corrosion region) and specimen B (uniform corrosion region) in the groundwater at 60 °C.

Figure 10 and Table 6 show the results of the potentiodynamic polarization test of specimen A and specimen B in the groundwater solution. The corrosion current density was controlled by the oxygen reduction reaction [31]. The corrosion current density was analyzed using the Tafel extrapolation method. Once the corrosion current density was determined, the corrosion rate can be calculated using the following equation [11]:

(1) $$\mathrm{Corrosion}\;\mathrm{rate}\;\left(\mathrm{mm}/\mathrm{year}\right)=0.00327\frac{a\cdot i_{corr}}{n\cdot D}\;$$

where a is the atomic weight, $i_{corr}$ is the corrosion current density, n is the number of equivalents exchanged, and D is the density of the low-carbon steel. Specimen A had a corrosion current density twice that of specimen B. The high corrosion rate in specimen A is due to the accelerated corrosion caused by Al inclusions and the larger pearlite phase fraction. In the vicinity of Al inclusions, localized corrosion occurs due to crevice corrosion, increasing the corrosion rate [19]. Larger pearlite phase fraction accelerates the corrosion rate via galvanic corrosion between pearlite and pro-eutectoid ferrite and between cementite and ferrite in pearlite [31]. In addition, when a larger fraction of pearlite exists around the Al inclusion, corrosion is accelerated by the larger fraction of pearlite, and aggressive ions, such as the Cl^−^ ion, are concentrated around the Al inclusion. This further accelerates crevice corrosion in Al inclusions. If there is no crevice around the Al inclusions, crevice corrosion does not occur. In addition, when pitting formed as crevice corrosion and galvanic corrosion progressed around the Al inclusions and the pearlite, the surface area became wider than the initial area due to morphological changes [29,35]. Equation (2) shows the anode current density according to the anodic overpotential in the activation polarization [11].

(2) $$i_{a}=i_{0}\exp\left(\frac{\alpha \cdot n\cdot F\cdot \eta_{a}}{2.3\cdot R\cdot T}\right)\;$$

In Equation (2), $i_{a}$ is the current density by the anodic overpotential, $i_{0}$ is the exchange current density, α is the fraction of $\eta_{a}$ taken by the ionization reaction, n is the number of equivalent exchanged, F is the Faraday’s constant, $\eta_{a}$ is the anodic overpotential, R is the gas constant, and T is the temperature. Equation (3) shows the correlation between area and current.

(3) $$i=\frac{I}{A}\;$$

In Equation (3), i is the current density, A is the reaction area, and I is the current. When the same overpotential was applied, the generated current (I) increased in proportion to the increased area. However, to obtain the current density, the reaction area (A) is equally divided by 1 cm^2^ for the generated current (I). Therefore, the change in roughness due to localized corrosion of specimen A causes higher current density on the potentiodynamic polarization curve.

### 3.7. Galvanostatic Polarization Test

**Figure 11:**
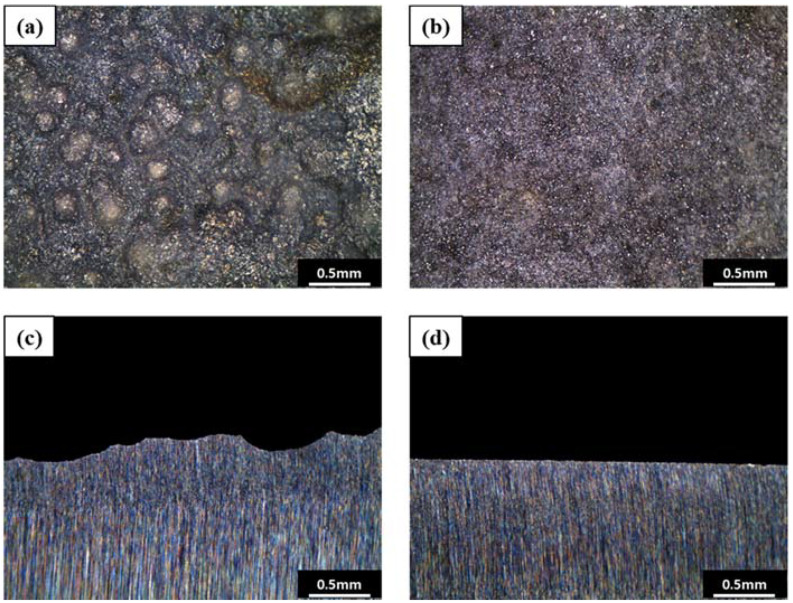
Surface and cross-sectional images of the specimens after galvanostatic polarization test: (**a**) surface image of specimen A (pitting corrosion region), (**b**) surface image of specimen B (uniform corrosion region), (**c**) cross-sectional image of the specimen A, and (**d**) cross-sectional image of the specimen B.

The galvanostatic polarization test was performed to accelerate corrosion. The acceleration time for the galvanostatic polarization test was calculated using the Faraday’s law as shown below [15]:

(4) $$i_{real}\cdot t_{real}=\frac{m\cdot F\cdot n}{a}=i_{accelerated}\cdot t_{accelerated}\;$$

where m is the reacted mass (g), i is the current density (A/cm^2^), t is the time (s), a is the atomic weight (g/mol), F is the Faraday’s constant (96,500 C/mol), and n is the number of electrons exchanged. The same coulombic charge was applied to observe the corrosion behavior for the same amount of corrosion. The total coulombic charge was 143.86 mAh, and the applied current was 5 mA/cm^2^. Figure 11 shows surface and cross-sectional images of specimen A and specimen B after galvanostatic polarization test. Pitting corrosion occurred in specimen A, and uniform corrosion occurred in specimen B. This indicates that pitting corrosion is related to the presence of Al inclusions and inhomogeneity of the pearlite.

### 3.8. Mechanism

**Figure 12:**
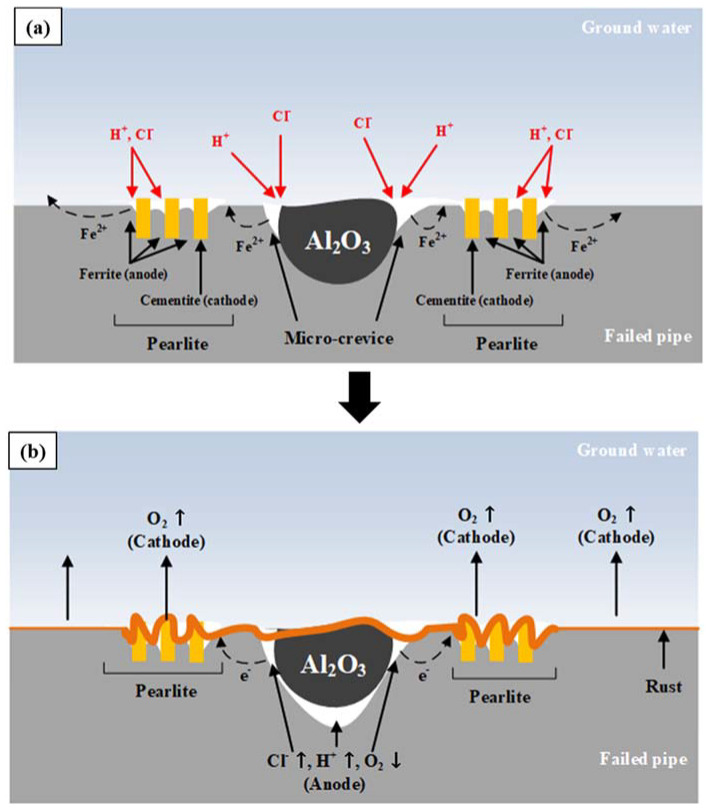
Failure mechanism of the failed low-carbon steel pipe based on aluminum inclusion and the larger phase fraction of the pearlite: (**a**) initial stage, and (**b**) later stage.

Figure 12 shows the failure mechanism of the failed low-carbon steel pipe due to the Al inclusions and a large amount of pearlite formed locally during the steelmaking process. Due to the coefficient of thermal expansion differences between Al and Fe, microcrevices form around Al inclusions during the pipe manufacturing process. As crevice corrosion is initiated, pH drops and Cl^−^ ions are concentrated in the microcrevice to maintain charge neutrality [19]. The concentration of Cl^−^ ions further accelerates crevice corrosion and corrosion products accumulate on this part of the pipe. Additionally, oxygen-concentration cells form, which accelerate localized corrosion, and eventually the Al inclusions fall off [19,29].

The large fraction of pearlite has a higher corrosion rate due to microgalvanic corrosion between the surrounding pro-eutectoid ferrite and pearlite, and between cementite and ferrite in pearlite [19,24]. Due to the relatively accelerated corrosion rate of the large fraction of pearlite near the Al inclusions, the concentration of Cl^−^ ions into Al vicinity and the accumulation of corrosion products are accelerated. This promotes the formation of an oxygen-concentration cell in the vicinity of the Al inclusion.

However, the presence of a large fraction of pearlite alone cannot cause localized corrosion such as pitting. The cementite inside the pearlite locally accelerates the surrounding ferrite corrosion by microgalvanic corrosion, but over time, the rust is covered by the dissolution of ferrite, and the corrosion proceeds in the form of a uniform corrosion [19]. In other words, a large fraction of pearlite accelerates corrosion locally, but cannot lead to pitting corrosion. That is, the large fraction of pearlite accelerates crevice corrosion via Al inclusions and promotes the formation of oxygen-concentration cell, thereby accelerating localized corrosion in the vicinity of the Al inclusions.

## 4. Conclusions

In this study, the failure analysis of a low-carbon steel pipe used in DH system was investigated using visual inspection, ICP-AES, OM, AFM, SEM/EDS, EPMA, and electrochemical tests. According to the results of the failure analysis, the following conclusions were drawn.

- Leakage occurred in the form of pitting corrosion, which was observed both inside and outside of the failed pipe. In particular, severe pitting corrosion occurred on the outside of the pipe, exposed to the soil environment. Al inclusions and a larger phase fraction of pearlite were observed near the leaking section. Crevice corrosion occurred in the microcrevice around the Al inclusions, and the large phase of pearlite around Al inclusions accelerated the localized corrosion in the microcrevice. Localized corrosion was accelerated near the Al inclusions and the large fraction of pearlite in the groundwater environment outside of the pipe, resulting in the pipe’s failure.
- The corrosion rate of the specimen taken where the pitting corrosion was present in the failed pipe was approximately double that of the specimen taken from the uniform corrosion part of the failed pipe. Furthermore, the corrosion type was similar to that observed in the actual failed pipe. This confirms the pipe failure had been caused by Al inclusions and the inhomogeneity of the pearlite.

## 5. Recommendations

- It is recommended that the uniform distribution of fine-sized pearlite and Al inclusions be produced through proper liquid steel homogenization and heat treatment during the steelmaking process.
- It is recommended that a standard for the chemical composition of Al be established within the existing KS D 3562 standard.

## Figures and Tables

**Table 1:** Chemical composition of district heating water (ppm) used in a district heating system.

pH	NaCl	Mg(OH)_2_	CaCO_3_	NH_4_OH
9.5	15.01	0.48	2.65	10.28

**Table 2:** Chemical composition of synthetic ground water (ppm).

pH	CaCl_2_	MgSO_4_∙7H_2_O	NaHCO_3_	H_2_SO_4_	HNO_3_
6.8	133.2	59.0	208.0	48.0	21.8

**Table 3:** Chemical compositions of the failed low-carbon steel pipe and KS D 3562 standard (wt. %).

Elements	C	Si	Mn	P	S	Al
Failed pipe	0.08	0.02	0.42	0.011	0006	0.04
KS D 3562	0.25	0.35	0.30–0.90	0.04	0.004	–

**Table 4:** Volume fraction of the pearlite and ferrite phases according to the specimen A (pitting corrosion region) and specimen B (uniform corrosion region) in the failed pipe.

	Pearlite (%)	Ferrite (%)
Specimen A	13.68 ± 0.58	86.32 ± 0.58
Specimen B	5.57 ± 0.34	94.43 ± 0.34

**Table 5:** Surface potential of different phase area and their differences.

Phase	Position	Potential (mV)		Surface Potential Difference (mV)
		Mean	Dev	
Pearlite	A	124.99	15.85	44.40
Ferrite	B	80.59	13.97	
Cementite in pearlite	C	147.22	13.56	34.78
Ferrite in pearlite	D	112.44	13.26	

**Table 6:** The electrochemical parameters resulting from the polarization measurements of specimen A and specimen B in the groundwater at 60 °C.

	*E_corr_* (mV_SCE_)	*i_corr_* (A/cm^2^)	Corrosion Rate (mm/yr)
Specimen A	−621.04	6.47 × 10^−^^5^	0.75
Specimen B	−704.63	3.33 × 10^−^^5^	0.39

## Data Availability

Not applicable.
